# The Relationship between *Mycoplasmas* and Cancer: Is It Fact or Fiction ? Narrative Review and Update on the Situation

**DOI:** 10.1155/2021/9986550

**Published:** 2021-07-31

**Authors:** Elhem Yacoub, Osama Mohammed Saed Abdul-Wahab, Mishari H. Al-Shyarba, Boutheina Ben Abdelmoumen Mardassi

**Affiliations:** ^1^Specialized Unit of Mycoplasmas, Laboratory of Molecular Microbiology, Vaccinology, and Biotechnology Development, Institut Pasteur de Tunis, University of Tunis El-Manar, Tunis, Tunisia; ^2^Department of Microbiology, Molecular Diagnostics Laboratory, Manchester Royal Infirmary, Manchester University NHS Foundation Trust, Manchester, UK; ^3^Department of Surgery, College of Medicine, King Khalid University, P.O. Box 641, Abha 61421, Saudi Arabia

## Abstract

More than one million new cancer cases occur worldwide every year. Although many clinical trials are applied and recent diagnostic tools are employed, curing cancer disease is still a great challenge for mankind. Heredity and epigenetics are the main risk factors often related to cancer. Although, the infectious etiological role in carcinogenesis was also theorized. By establishing chronic infection and inflammation in their hosts, several microorganisms were suggested to cause cell transformation. Of these suspicious microorganisms, mycoplasmas were well regarded because of their intimate parasitism with host cells, as well as their silent and insidious role during infections. This assumption has opened many questions about the real role played by mycoplasmas in oncogenesis. Herein, we presented a sum up of many studies among the hundreds which had addressed the *Mycoplasma*-cancer topic over the past 50 years. Research studies in this field have first started by approving the mycoplasmas malignancy potential. Indeed, using animal models and in vitro experiments in various cell lines from human and other mammalians, many mycoplasmas were proven to cause varied modifications leading to cell transformation. Moreover, many studies have looked upon the *Mycoplasma*-cancer subject from an epidemiological point of view. Diverse techniques were used to assess the mycoplasmas prevalence in patients with cancer from different countries. Not less than 10 *Mycoplasma* species were detected in the context of at least 15 cancer types affecting the brain, the breast, the lymphatic system, and different organs in the genitourinary, respiratory, gastrointestinal, and urinary tracts. Based on these revelations, one should concede that detection of mycoplasmas often linked to ‘‘wolf in sheep's clothing” is not a coincidence and might have a role in cancer. Thorough investigations are needed to better elucidate this role. This would have a substantial impact on the improvement of cancer diagnosis and its prevention.

## 1. Introduction

Cancer is a devastating disease presenting an immense burden to humanity. According to latest statistics of the International Agency for Research on Cancer affiliated to the World Health Organization, barely 18 million new cancer cases were globally recorded in 2018, of which approximately 10 million have led to death [1]. Many causes and risk factors have been pointed to promote carcinogenesis establishment and development. Among these factors, some infectious agents have been suspected. In fact, according to the American Cancer Society, up to 20% of cancers worldwide have been related to infectious agents. The causal relationship between different types of cancer and many oncoviruses such as papillomavirus, hepatitis B virus, and Epstein-Barr virus and bacteria such as *Streptococcus bovis*, *Salmonella typhi*, *Chlamydia pneumoniae*, *Bartonella*, and *Helicobacter pylori* has previously been well documented [2–12]. One of the suspected prokaryotes in malignancy are mycoplasmas. As these atypical bacteria are notorious for their capacity to implement a low-grade chronic inflammatory condition during cell infection without compromising viability, they were thought to be ideal for promoting cancer transformation [13]. Actually, the oncogenic potential and role of mycoplasmas in cancer development have been started to be investigated since the 1950s. Mycoplasmas were first detected in leukemia patients, and later, many studies have reported their identification in variant solid cancers either directly by PCR amplification of specific DNA segments or indirectly by evaluation of their antibody status in patients. Throughout this review, we will attempt to highlight the *Mycoplasma*-cancer relationship. Twenty years from the first review describing this relationship [14] and ten years after the second [15], we are today recalling what has been performed in this regard along with the new findings.

## 2. Strategy Research

We have conducted this review by synthesizing the results of research studies that consider *Mycoplasma* as an infectious agent involved in cancer. This was done by summarizing the available data with regard to the impact of mycoplasmas on the morphology, properties, and signaling pathways in mammalian cells and listing the different cases of cancer in which *Mycoplasma* species have been identified.

This review has been elaborated on the basis of screening in PubMed/MEDLINE databases, all types of published articles (research articles, reviews, and case reports) which have approached the *Mycoplasma*-cancer topic. This synthetic study was further complemented and refined by scanning the reference lists of the selected articles. All the bibliographical sources included in this review were carefully consulted and approved by all the authors.

In addition to the introduction and the conclusion, the main body of this review was conceived in three sections. The first is devoted to a general and concise description of distinctive features of mycoplasmas that made them suspicious infectious agents in cancer. To prove their malignancy potential, we have drawn on 27 studies, the synthesis of which constituted the content of the second section. The third section includes the first seven studies published between 1965 and 1970, which reported the presence of mycoplasmas in cancer patients, and 27 more recent studies published between 1995 and 2020. These have provided epidemiological information on the identified mycoplasmas in many types of cancer in several countries.

## 3. Mycoplasmas: Atypical Tiny Microorganisms Capable of Causing Serious Troubles

Mycoplasmas are atypical bacteria widespread in nature as parasites of human, mammals, reptiles, fish, birds, arthropods, and plants [16]. Mycoplasmas were first thought to be viruses given their reduced genome, minute size, and the total lack of a cell wall around their cytoplasmic membrane. They are considered as the smallest self-replicating prokaryotes [17]. The extensive genome reduction, through a process of reductive evolution, has limited the mycoplasmas biosynthetic capacity and hampered their metabolic pathways [18, 19]. As a result, mycoplasmas are rendered completely dependent on their hosts to acquire essential precursors such as nucleotides and amino acids to insure their survival [20–23]. Mycoplasmas have tropism for many types of eukaryotic cells, and most of them are extracellular. However, host cell invasion was reported for some *Mycoplasma* species such as *Mycoplasma fermentans* and *Mycoplasma penetrans* [24, 25].

Despite their genetic deficiency, mycoplasmas are able to cause diseases of significant economic impact, especially in livestock animals [26–29]. For human, mycoplasmas have been also associated to many serious pathologies such as respiratory troubles [30–33], urogenital issues [34–37], infertility [38–41], rheumatic diseases [42–44], and AIDS [45–49]. These diseases are usually caused by acute mycoplasma infections. However, some mycoplasmas are capable to chronically colonize human cells without obvious clinical symptoms. Being a cell wall-free prokaryote, mycoplasmas interact closely with mammalian cells in silence for long time. This mute and extended interaction could be at the origin of alteration of many biological characteristics of mammalian cells [50]. Therefore, a potential association between mycoplasmas and cellular malignancy was suggested. The investigation of this oncogenic potential has been started from the middle of the 1960s, when several studies reported the detection of some human *Mycoplasma* species or their antibodies in patients suffering from leukemia [51–57]. Although these studies did not provide causal evidence for the involvement of mycoplasmas in cancer, they represented an opportunity for further investigations.

## 4. Malignancy Potential of Mycoplasmas: Evidence In Vitro and In Vivo

After the first reports suggesting the existence of a relationship between mycoplasmas and cancer, many experiments have subsequently been carried out to determine whether these bacteria are really endowed with oncogenetic properties or they are fortuitly identified in patients diagnosed with cancer. Other studies have attempted to explore whether mycoplasmas are directly involved in the onset of cancer or rather in its progression. Many cellular and molecular mechanisms of malignancy transformation were proposed for mycoplasmas.

For example, Paton et al. have shown that following serial passages of human diploid WJ-38 stem cells from normal embryonic lung and their infection with *Mycoplasma orale*, the growth of these cells regresses with noticeable chromosomal aberrations. This is the first study reporting such chromosomal disorders associated with *Mycoplasma* infection in normal human cells [58]. Malignant transformation induced by mycoplasmas was also reported to occur in blood cells (PBMCs) [59] and in many other human cell lines from different organs such as leiomyosarcoma cell line SK-UT-1B in the uterus [60], adenocarcinoma A549 cells in the lung [61], BPH-1 cells in the prostate [62], and neuronal cell line BE-M17 [63]. Besides human cells, studies have also demonstrated that some *Mycoplasma* species are effective in mediating changes in other mammalian cells in mouse, monkey, and hamster. The time of inoculation and the passage level were shown to affect transformation of infected mammalian cells [64, 65]. These findings were consolidated by another similar study fulfilled by Tsai et al., by exploring the impact of infection with *M*. *fermentans* and *M*. *penetrans* species on mouse embryo cells C3H. In this study, a *Mycoplasma*-mediated oncogenesis was proved to take place in progressive steps and not in an acute way. In reality, the phenotypic changes noticed become progressively more conspicuous with the persistence of the *Mycoplasma* infection [13]. These two *Mycoplasma* species were also shown, in another study, to infect the murine 32D hematopoietic cell line, which is known to undergo interleukin-3-dependent apoptosis. It was found that mycoplasmas infection altered the properties of the 32D cells, preventing their apoptotic capacity, and enabling them to grow autonomously, independently of interleukin-3 stimulation. This was evidenced by the formation of tumors after injection of transformed 32D cells into nude mice [50]. It has also been found that the properties of stem cells are affected and altered by *Mycoplasma* infection [66].

Some researchers have been rather interested in studying the impact of activation or inactivation of *Mycoplasma* infections on the expression of target genes in different cells. These targets included oncogenes, tumor suppressors, proinflammatory cytokines, and growth factors. Zhang et al. have reported that chronic infection with the two human *Mycoplasma* species *M*. *fermentans* and *M*. *penetrans* is associated with an overexpression of H-*ras* and c-*myc* oncogenes in C3H embryo cells, which involved malignant cell transformation in mouse [67]. Subsequently, other scientific teams from the same laboratory have examined and compared the expression profiles of many genes (38 cytokine genes and 1185 other genes involved in oncogenesis, apoptosis, cell growth, and cell cycle regulation) in different human and mouse cells, before and after infection with different urogenital mycoplasmas. They concluded that distinctive mycoplasmas may have different effects (sometimes opposite) on the expression of a given gene. These effects might progressively but significantly alter several biological properties of mammalian cells (obvious changes in cell morphology or growth rate) and thus lead to malignant transformation [68, 69]. Using different approaches, Liu and Shou have also demonstrated that *Mycoplasma* infection has several effects on mammalian cells. These include genome breakdown and dysregulation of the expression of certain genes involved in apoptosis and tumorigenesis [70]. Furthermore, the repercussion of mycoplasmas infection on major regulation mechanisms of programmed cell death was also investigated. Logunov et al. have monitored the effects of *M*. *fermentans*, *M*. *arginini*, *M*. *hominis*, and *M*. *arthritidis* on the p53 tumor suppressor and the nuclear factor kappaB (NF-*κ*B) pathways, both involved in the maintenance of the cell cycle stability. Experiments carried out in vitro on a panel of human and mice cell lines showed that *Mycoplasma* infection inhibited p53 activity and activated the NF-*κ*B, which are specific traits of human tumor cells. Moreover, it has been found that the same *Mycoplasma* species mentioned above cooperates with some oncogenes in cell transformation. However, the involved *Mycoplasma* protein has not been identified [71]. This was not the case in other studies identifying a *M*. *hyorhinis*-specific protein, p37, which promotes the invasiveness of the pathogen into the human cells. This demonstrates its involvement in the malignancy process [72–74]. A significant association between this *Mycoplasma* species and tumorigenesis was once again proved in vitro and in vivo in another study [75]. This was further strengthened by two more recent research studies supporting the role of *M*. *hyorhinis* in malignant transformation of gastric cells. This is achieved either by the activation of the NLRP3 inflammasome, which mediates the maturation of proinflammatory cytokines [76] or by activating the *β*‐catenin signaling pathway, which is crucial for tissue homeostasis [77]. Furthermore, another way for the implication of mycoplasmas in cancer promotion was proposed. Mycoplasmas were shown to disturb the anticancer process either by causing resistance to the anticancer drugs or by repression of the natural killer cells activity. In two studies realized by two teams from the University of Maryland in the USA, the *Mycoplasma* chaperone DnaK was shown to have oncogenetic properties that render it responsible for the potency decrease of some proteins associated with critical cellular pathways, leading to a decline in the efficiency of anticancer drugs [78, 79]. Likewise, a group of Russian and German scientists have reported that sensitivity of tumorous cells to anticancer decreases after *Mycoplasma* infection. The authors have precisely worked with *M*. *hyorhinis* species [80]. In a Singaporean-Taiwanese study that came out just since few months, *Mycoplasma* infection was used to give rise to an artificial chronic inflammation condition in order to understand the influence of macrophages on natural killer cells in a context of a cancer. Results have shown that cancerous tissues infected by mycoplasmas were protected by macrophages from the attack of natural killer. Mycoplasmas have served as an example to prove that infection-induced inflammation helps in the cancer progression through natural killer cell repression moderated by macrophages [81].

**Figure 1:**
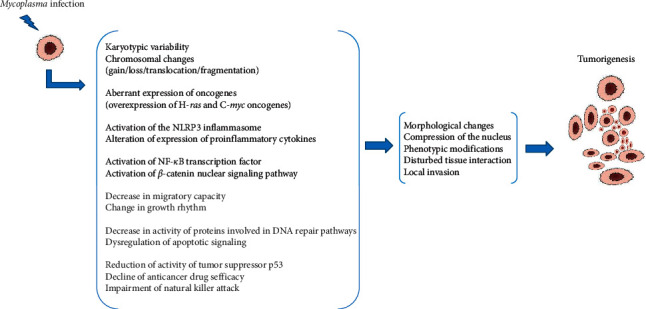
Modifications in *Mycoplasma*-infected cell lines leading to carcinogenesis.

As reported above, both in vitro and in vivo experimental data provided evidence that some mycoplasmas are able to affect the accuracy of genomic transmission during cell division and to disturb the coordination between cell cycle checkpoints. Many cases of chromosomal abnormalities (loss and translocations), karyotypic changes, and morphological modifications have been associated to *Mycoplasma* infections. More details about the above selected experimental studies are given in Table 1. As well, a schema assembling the different effects caused by *Mycoplasma* infections on cell lines leading to malignant transformation is presented in Figure 1.

## 5. Cancer-Mycoplasmas Relationship from an Epidemiological Standpoint: Overview of the Reported Cases

After the above listed studies, admitting the malignancy potential of mycoplasmas, several epidemiological investigations based on molecular and serological detection of these bacteria in different types of cancers have been documented in many countries around the world (Table 2).

### 5.1. Prostate Cancer

Prostate cancer is the second most commonly diagnosed cancer and the sixth cause of cancer death in men worldwide. Incrimination of urogenital mycoplasmas in this type of cancer has been evoked since the mid-twentieth century [110, 111]. Based on this suggestion, some studies have been conducted later to experimentally prove the mycoplasmas potential in malignant transformation of prostate cells [62, 73]. More recently (during the last 2010–2020 decade), this hypothesis was further sustained by several studies reporting the detection of mycoplasmas (by PCR or RT-PCR) or their antibodies (using ELISA technique) in patients suffering from prostate cancer. Different *Mycoplasma* species, notably *M*. *hominis*, *M*. *genitalium*, and *Ureaplasma urealyticum*, were identified with variable percentages in patients from different nationalities such as Russian, Turkish, Australian, Iranian, and Japanese [82–87]. In the same context, our team has recently shown the presence of *U*. *parvum* and *U*. *urealyticum* species in a cohort of Saudi patients diagnosed with prostate cancer with rates of 20% and 16%, respectively [88].

It is important to notice that although the epidemiological data reported in these studies are interesting and persuasive, a luck of statistical and/or experimental evidences in some of them constitutes a limiting factor in the approving of the etiopathological role of mycoplasmas in prostate cancer.

### 5.2. Gastric Cancer

Malignant transformation in stomach cells is also one of the most frequently occurring cancers worldwide. In addition to the risk factors often mentioned in stomach cancer, such as smoking and lack of healthy nutrition, *Helicobacter pylori* infection was well established as a cause of this cancer. Indeed, it is the most and best studied case illustrating the direct involvement of an infectious agent in cancer [10–12]. This has encouraged further investigations to determine the potential involvement of other infectious agents in this type of cancer such as mycoplasmas. Results of studies aiming to seek for *Mycoplasma* species in patients with gastric cancer have shown the frequent detection of *M*. *hyorhinis*. This *Mycoplasma* species was identified by the Southern blot technique using specific *Mycoplasma* rDNA probes in 11 out 23 Japanese patients with gastric cancer (48%) [89]. Higher detection rates around 56% [90] and 54.1% [91] were reported in tumors from Chinese patients using the immunohistochemistry assay. A higher rate of 63.9% was obtained with nested PCR [92]. Interestingly, this last study also reported the detection of another *Mycoplasma* species, *M*. *fermentans*, however, always in conjunction with *M*. *hyorhinis* in the recruited patients. The authors of this study even succeeded in culturing these species on some cancerous specimens. This constitutes an irrefutable evidence of their existence in cancerous tissues.

### 5.3. Ovarian Cancer

According to the Global Cancer Observatory, nearly 300 thousand new cases of ovarian cancer were recorded in 2018. With about 185 thousand deaths, this type of cancer is considered one of the most deadly of the gynecological cancers [1]. Several risk factors contribute to the development of this type of cancer, the genetic predisposition being the most suspected [107, 112]. However, the involvement of some urogenital microorganisms, such as *Chlamydia trachomatis*, in the etiology of ovarian cancer has also been raised. Such a suggestion has been investigated on the basis of the frequent detection of *Chlamydia trachomatis*, which is marked by a persistent infection in the female upper genital tract [113, 114]. Endowed with the same properties, mycoplasmas were also suspected and their association with ovarian cancer was assessed in some studies. Indeed, in one of the investigations carried out, a combined PCR-ELISA method was designed for the detection of 15 *Mycoplasma* species in women with ovarian cancer. *Mycoplasma* infection was identified in more than half of 27 patients tested, which is a significant rate of about 60% [93]. Using the same combined procedure, Kim et al. found a rate of only 13.8% (4 among 29 Korean patients with ovarian cancer) that harbor *Mycoplasma* DNA [94]. Another study reported a rate of only 13% (6 samples out of a total of 46) of mycoplasmas in ovarian cancerous tissue using the nested PCR technique. Sequencing and BLAST analysis of the six positive cases identified *M*. *salivarium* and *M*. *arginini*. Given the low detection rate and the contaminating nature of the identified *Mycoplasma* species, the authors stated that their results are insufficiently consistent to confirm any relationship between *Mycoplasma* and ovarian cancer [95]. Idahl et al. undertook a similar work, but focused on searching for a particular species of *Mycoplasma*, *M*. *genitalium*. Their results revealed the presence of antibodies to *M*. *genitalium* in patients with ovarian cancer. But the statistics and comparison with controls showed that the association between this *Mycoplasma* species and ovarian cancer is not significant [96]. Overall, *Mycoplasma* detection rates in women with ovarian cancer do not seem to support the hypothesis that these microorganisms are involved in ovarian cancer. Larger epidemiological studies and more sophisticated methods are needed to better define the role of mycoplasmas in the tumorigenesis of ovarian tissue.

### 5.4. Cervical Cancer

Cervical cancer is one of the most common cancers among women worldwide, particularly in the low- and middle-income countries. Together with breast cancer, it is classified at the top of the most commonly diagnosed cancers and the leading causes of death in women [1]. Some carcinogenic microorganisms such as the human papillomavirus and the human immunodeficiency virus were identified as a cofactor for the development of cancerous lesions in the uterine cervix [115–117]. Because they are commonly detected in the cervical microbiome, mycoplasmas have brought the attention of some scientists who have eventually detected them in women with cervical cancer. Some *Mycoplasma* species were identified, notably, *M*. *penetrans*, *M*. *genitalium*, *M*. *hominis*, and *U*. *urealyticum* [97–99].

### 5.5. Other Types of Cancer (Colon, Esophagus, Breast, Brain, Lymphatic System, Kidney, Bladder, Larynx, Tongue, and Lung)

In their study evaluating *Mycoplasma* infections in different types of cancer in humans, Huang et al. have communicated interesting results showing that *Mycoplasma* infection might occur in tumor cells in different organs such as the colon, esophagus, breast, and brain [90]. A tumor affecting the lymphatic system (non-Hodgkin's lymphoma) was also suspected to be related to *Mycoplasma* infection. Indeed, *M*. *fermentans* was incriminated [100]. The relationship between *Mycoplasma* infection and renal carcinoma has also been investigated. In two different studies, similar high detection rates, exceeding 80% of mycoplasmas DNA have been found in tissues of cancer patients. Such interesting results prompted the authors to consider the clinical significance of *Mycoplasma* infection in patients suffering from kidney cancer [101, 102]. Likewise, the development of cancer in bladder cells was previously associated with mycoplasmas. *M*. *penetrans* was more involved in the development of bladder cell cancer [103].

Mycoplasmas have been detected in the oral cavity and in the upper and lower respiratory tracts. Baracaldo et al. reported the case of a man with cancer of the larynx, presenting empyema probably caused by *M*. *salivarium*. The suspicion of mycoplasmas occurred following the observation of the growth of small Gram-negative colonies in the pleural fluid collected from that patient. No other bacteria or fungi were detected in the sample. PCR followed by sequencing identified *M. salivarium* [104]. The same species was also detected in a tumor in a patient with squamous cell carcinoma of the tongue [105]. Another study reported the case of a young woman with a cervical squamous cell carcinoma probably related to oropharyngeal infection by *M*. *hominis* species [106]. Moreover, patients with lung cancer were also found to harbor mycoplasmas. Indeed, Huang et al. have communicated a relatively high detection rate about 52.6% for mycoplasmas in Chinese patients diagnosed with lung cancer [90]. Pehlivan et al. conducted the same research but found a *Mycoplasma* rate of 22.2% in patients with lung cancer [108]. In a more recent study presented during the CHEST annual meeting held in Canada in 2017, Reddy et al. reported the detection of *M*. *pneumoniae* IgM in the serum of an elderly woman diagnosed with adenocarcinoma of the lung [109].

## 6. Conclusion

Several studies have raised concern over *Mycoplasma*-cancer subject from different perspectives. The purpose of this article was to review the experimental and epidemiological studies on the possible association between cancer and mycoplasmas. Despite the multiple studies approving that mycoplasmas are endowed with a malignancy potential and those reporting data about their prevalence in patients with different types of cancer, the etiopathological role of mycoplasmas in tumorigenesis is still controversial and debatable. Indeed, the association between *Mycoplasma* and cancer has been often put in doubt as some studies did not report sufficient evidences on mycoplasmas' oncogenic properties [118] nor on their ability to induce transformation [119]. Aside of the repetitive detection of *M*. *hyorhinis* in gastric cancer, no other obvious specific association may be inferred between a given *Mycoplasma* species and a particular type of cancer. Nevertheless, the detection of mycoplasmas antibodies remains an efficient mean of monitoring their infection in patients with cancer and could provide evidence of their presence and therefore of their possible involvement. Although PCR amplification is the most useful and direct way to detect mycoplasma DNA, the in situ localization by culture remains the best one to confirm the presence of live and active mycoplasmas in tumor sites. This supports the hypothesis of causality for the involvement of these bacteria in host cells transformation.

We cannot ignore the vulnerability to infectious agents, including mycoplasmas, of an immunodeficient organism subsequent to cancer. To exclude such a case, further investigations should be undertaken to provide strong and irrefutable proof of a causal relationship between mycoplasmas and cancer and to elucidate the mechanisms governing their potential for malignancy. In order to do so, researchers should ask a question: could the cancers associated with infectious agents be avoided if the infection in cause is prevented? If it is shown that reliable diagnosis and early prevention of infection will prevent the development of cancer, this would strengthen the theory of the infectious origin of cancers.

## Figures and Tables

**Table 1:** In vitro and in vivo studies indicating malignant potential of mycoplasmas.

*Mycoplasma* species	Cell line	Observed effect	Country	Year	Reference
*M*. *orale*	Human diploid cell strain WJ-38 from normal embryo lung	(i) Fragmented chromosomes (ii) High level of breaks and polyploidy	UK	1965	[58]
*M*. *hominis* *M*. *fermentans* *M*. *orale* *M*. *pneumoniae* *M*. *salivarium*	BHK2I line of hamster fibroblasts	(i) Cell growth with altered colonial morphology	UK	1966	[65]
*Spiroplasma pirum*	Mouse NIH 3T3 cells Monkey kidney CV-1 cells	(i) Morphological changes of cells (ii) Growth on soft agar and tumors development in athymic and BALB/*c* mice	USA	1986	[64]
*M. hyorhinis*	Mouse FS9 sarcoma cells	(i) Reduction of invasiveness of transformed cells, approved by using specific monoclonal antibody directed against *M*. hyorhinis-specific protein p37	Switzerland	1990	[72]
*M*. *fermentans* *M*. *penetrans*	Mouse embryo C3H cells	(i) Phenotypic changes of infected cells (ii) Ability to form tumors in animals (iii)High soft agar cloning efficiency	USA	1995	[13]
*M. fermentans* *M. penetrans*	Mouse embryo C3H cells	(i) Multistage malignant transformation (ii) Induction of H-ras and c-myc oncogenes expression (iii) Morphological changes and uncontrolled cell growth	USA	1997	[67]
*Acholeplasma laidlawii*	Human uterine leiomyosarcoma cell line SK-UT-1B	(i) Chromosomal aberrations (ii) Karyotypic variability	Russia	1998	[60]
*M*. *fermentans* *M*. *penetrans* *M. salivarium* *M. genitalium* *M. pneumoniae* *M. orale* *M. pirum*	Murine hematopoietic 32D cell line	(i) Activation of NF-Κb transcriptiontranscrption factor (ii) Continuous growth without interleukin stimulation (iii) Chromosomal changes and trisomy 19	USA	1999	[50]
*M*. *fermentans M*. *genitalium* *M*. *hominis* *M*. *penetrans*	Immortalized cervical and prostatic epithelial cells	(i) Alteration in expression of cytokine genes (more profoundly in cervical epithelial cells) - (ii) radually alterationaltereration of important biological properties	USA	2000	[69]
*M*. *fermentans*	Human peripheral blood mononuclear cells (PBMCs) from healthy blood donors	(i) Karyotype change (ii) Chromosomal loss or gain and translocation	USA	2004	[59]
*M. fermentans*	Mouse embryo C3H cells	(i) Aberrant expression of many oncogenes and tumor repressors at different infection stages	USA	2006	[68]
*M. hyorhinis*	Prostate cancer PC-3 and DU145 cell lines	(i) Invasion of cancer cells (ii) Nuclear enlargement (iii) Anaplastic cells (iv) Increase of migratory potential (v) Changes in growth rhythm (vi) Changes in morphology and gene expression	USA	2007	[73]
*Mycoplasma* spp.	Different types of cell lines (mesenchymal, epithelial, and myeloid) Immortalized human bronchial epithelial BEAS-2B cells Lung adenocarcinoma A549 cells	(i) Cell transformation, proliferation, differentiation, a and apoptosis	USA	2008	[61]
*M. fermentans* *M. arginini* *M. hominis* *M. arthritidis*	H1299, MCF-7, HCT116, BJ, REF52, 293 cells	(i) Activation of NF-*κ*B (ii) Inhibition of p53 pathway activity (iii) M. arginini showed the strongest effect among the tested species (iv) Cooperation with some oncogenes in cell transformation	Russia USA	2008	[71]
*M*. *genitalium* *M*. *hyorhinis*	Benign human prostate cells (BPH-1)	(i) Malignant transformation (ii) Formation of xenograft tumors in athymic mice (iii) Increase in karyotypic entropy (iv) Accumulation of chromosomal aberrations and polysomy	USA	2009	[62]
*M. hyorhinis*	Human MGC803 gastric carcinoma cells	(i) Elevation of tumor cell migration, invasion, and metastasis	China	2010	[75]
*M. fermentans* *M*. *hyorhinis*	Immortalized 32D and COS-7 cell lines HeLa and AGS tumor cell lines	(i) Growth inhibition of immortalized cell lines and tumor cell lines (ii) Compression of the nucleus (iii) Cell genome degradation (iv) Dysregulation of the expression of genes related to proliferation, apoptosis, tumorigenesis, and signaling pathway	China	2011	[70]
*M. hyorhinis*	Gastric cancer cell MGC803 THP-1 cells PMA-induced macrophages Human monocytes isolated from human peripheral blood mononuclear cells (PBMCs)	(i) Malignant transformation *of gastric cancer cells* (ii) *Activation of the protein complex* NLRP3 inflammasome, controlling the maturation of proinflammatory cytokines	China	2013	[76]
*M. hyorhinis*	Gastric cancer cell AGS Gastric cancer cell MGC803 Immortalized human gastric epithelia GES-1 Primary human fetal colon fibroblasts CC-18Co Human umbilical vein endothelial cell (HUVEC) Primary mouse embryonic epithelia (MEF) Primary mouse gastric epithelia (PMGE)	(i) Promotion of gastric cancer cell invasiveness (ii) Activation of NF-*κ*B pathway	China	2014	[74]
*Mycoplasma* spp.	LM-Mel-14, 24, and 34 cell lines LM-Mel-53, 56, 62, and 93 cell lines de novo established from patient tumors in stem cell media	(i) Damage and alteration of stem cells properties (clonogenicity, proliferation, and global gene expression) (ii) Expression of putative cancer stem cell markers	Australia USA	2016	[66]
*M. fermentans*	Colon carcinoma HCT116 cell line Female NOD/SCID and NOD/SCID-*γ* (NSG) mice	(i) Lymphomagenesis in SCID mice (ii) Decrease in the activity of proteins involved in cellular pathways required for DNA repair (iii) Impair of anticancer functions (iv) Reduction of anticancer drugs efficacy (v) *M*. *fermentans* chaperone protein DnaK (heath shock protein) was proved to cause these oncogenetic properties	USA	2018	[79]
*M. hyorhinis*	Human lung mucoepidermoid carcinoma NCI-Н292 cells	(i) Decrease of sensitivity of tumerous cells to anticancer (nutlin-3) (ii) Infected cells were more capable of growing and dividing	Russia	2018	[80]
*Mycoplasma* spp.	Dopaminergic neuronal BE-M17 cell line	(i) Induction of oxidative stress with no inflammatory response (ii) DNA damage (iii) Decrease in the efficiency of pathways necessary for DNA repair	USA	2019	[63]
*M. hyorhinis*	Human gastric cancer MGC803 and BGC823 cell lines	(i) Accumulation of nuclear signaling pathway *β*‐catenin Enhancement of the expression of downstream *β*‐catenin genes (ii) Induction of gastric cancer cell motility	China	2019	[77]
*M*. *fermentans*	HCT116 cells transfected with expression vector containing *M*. *fermentans* DnaK	(i) DnaK protein plays a critical role in pathways involved in recognition of DNA damage and repair (ii) Reduction of p53 stability and anticancer functions	USA	2020	[78]
*Mycoplasma spp.*	Chronic myeloid leukemia (CML) K562 and KCL-22 cell lines Macrophages Natural killer	(i) Cancerous tissues infected by mycoplasmas were protected by macrophages from the attack of natural killer	Singapore Taiwan	2020	[81]

**Table 2:** Data from epidemiological studies performed in the context of *Mycoplasma*-cancer relationship.

Cancer type	Method	*Mycoplasma* species	Detection rate (%)^*∗*^	Study population^*∗*^	Country	Year	Reference
Prostate cancer	RT-PCR	*M*. *hominis*	15	125	Russia	2011	[82]
	ELISA	*M*. *hominis*	3	118			
	PCR	7 *Mycoplasma* spp.	35.4	31	Turkey	2013	[83]
	RT-PCR	*M*. *genitalium*	9.56	115	Australia	2014	[84]
		*U*. *urealyticum*	0.86				
		*U*. *parvum*	0				
	PCR	*U*. *urealyticum*	1.61	62	Iran	2015	[85]
		*M*. *genitalium*	0				
	PCR	*M*. *genitalium*	40	45	Japan	2019	[86]
		*M*. *hyorhinis*	0				
		*U*. *urealyticum*	0				
	RT-PCR	*M*. *hominis*	13.11	61	Iran	2020	[87]
	PCR and sequencing	*U*. *parvum*	20	50	KSA	2020	[88]
		*U*. *urealyticum*	16				

Gastric cancer	PCR	12 *Mycoplasma* spp.	48	23	Japan	1995	[89]
	Southern blot	*M*. *hyorhinis*	15	27			
	Immunohistochemistry PCR	*M*. *hyorhinis*	56	90	China	2001	[90]
	Immunohistochemistry	*M*. *hyorhinis*	54.1	98	China	2002	[91]
	Immunohistochemistry	*M*. *hyorhinis*	45.9	61	China	2006	[92]
		*M*. *fermentans*	—				
	Nested PCR	*M*. *hyorhinis*	63.9				
		*M*. *fermentans*	31.14				
	Culture	*M*. *hyorhinis*	8.19				
		*M*. *fermentans*	3.27				
		*M*. *hyorhinis* *+* *M*. *fermentans* coinfection	1.63				

Ovarian cancer	Combined PCR-ELISA	15 *Mycoplasma* spp.	59.3	27	USA	1996	[93]
	Combined PCR-ELISA	15 *Mycoplasma* spp.	13	29	Korea	1998	[94]
	Nested PCR	12 *Mycoplasma* spp.	13	46	USA	2001	[95]
	Sequencing	*M*. *arginini*	5				
		*M*. *salivarium*	1				
	Lipid-associated membrane protein-enzyme immunoassay (LAMP-EIA)	*M*. *genitalium*	11.76	68	Sweden	2011	[96]

Cervical cancer	Combined PCR-ELISA	15 *Mycoplasma* spp.	33.3	9	USA	1998	[97]
	Nested PCR	*M*. *penetrans*	45.45	55	China	2007	[98]
	Metagenomic sequencing	Ureaplasma spp. M. hominis M. genitalium	51.4 34 2.3	134	USA Tanzania	2020	[99]

Colon cancer	Immunohistochemistry PCR	*Mycoplasma* spp.	55.1	58	China	2001	[90]
Esophagus cancer			50.9	53			
Breast cancer			39.7	63			
Brain cancer			41	91			

Non-Hodgkin's lymphoma	PCR	*M*. *fermentans*	10.9	265	UK	2001	[100]
Renal cancer	Nested PCR	15 *Mycoplasma* spp.	82	33	Turkey	2005	[101]
	Nested PCR	*Mycoplasma* spp.	81.8	95	China	2008	[102]
Bladder cancer	Culture and nested PCR	*M*. *penetrans*	41.8	55	China	2009	[103]
Laryngeal cancer	Culture and PCR	*M*. *salivarium*	—	1 case	USA	2012	[104]
Tongue cancer	TaqMan PCR	*M*. *salivarium*	—	1 case	Germany	2014	[105]
Oropharynx cancer	PCR	*M*. *hominis*	—	1 case	France	2020	[106]

Lung cancer	Immunohistochemistry PCR	*Mycoplasma* spp.	52.6	59	China	2001	[107]
	PCR	*Mycoplasma* spp.	22.2	27	Turkey	2004	[108]
	ELISA	*M*. *pneumoniae*	—	1 case	Canada	2017	[109]

^*∗*^Detection rate in control populations is not reported here. Only patients with confirmed cancer diagnosis are considered. (—) sign, data not mentioned.

## Data Availability

The data used to support the findings of this study are included within the article.
